# Exploring the Impact of Nickel Exposure on Female Fertility

**DOI:** 10.7759/cureus.57681

**Published:** 2024-04-05

**Authors:** Marc Ganz, Shoshana Devor, Yehuda Gejerman, Tzipora Benyaminov, Yaakov Ishakis, Moshe Bulmash, Daniel Miller

**Affiliations:** 1 Public Health Sciences, State University of New York Downstate Health Sciences University, New York City, USA; 2 Obstetrics and Gynecology, State University of New York Downstate Health Sciences University, New York City, USA; 3 Internal Medicine, New York Medical College, Valhalla, USA; 4 Internal Medicine, Icahn School of Medicine at Mount Sinai, Queens Hospital Center, New York City, USA

**Keywords:** family planning, general obstetrics, toxic substances, nickel, fertility

## Abstract

Introduction

Infertility affects an estimated 186 million individuals globally and is associated with numerous mental health issues. Trace elements are essential for reproductive health, yet the role of nickel in female fertility is not well understood. Previous research has shown conflicting evidence regarding nickel's impact on ovarian function and its potential to disrupt reproductive processes.

Methods

We utilized data from the National Health and Nutrition Examination Survey (NHANES) focusing on a cohort of 7,839 women, with an emphasis on 1,404 women aged 18 to 35. Logistic regression was employed to investigate the relationship between urinary nickel levels and fertility status, controlling for age, BMI, and race/ethnicity.

Results

The fertility analysis included 880 fertile and 106 infertile women, identifying age as a significant predictor of fertility status. Nickel exposure did not demonstrate a statistically significant association with fertility. The racial distribution within the groups showed a higher proportion of non-Hispanic White women in the fertile category and Mexican American women in the infertile group, although race was not a significant predictor in the regression model.

Conclusions

We found no significant relationship between nickel exposure and fertility status when adjusted for race, BMI, and age. Age was the only significant factor associated with fertility. These results highlight the complexity of the interplay between environmental exposures and reproductive health, suggesting that further research is necessary to elucidate the role of nickel and other trace elements in fertility.

## Introduction

Infertility, defined by the World Health Organization as the inability of a couple to conceive despite regular, unprotected sexual intercourse for over a year, affects an estimated 186 million individuals globally [1,2]. It poses significant mental health challenges, including anxiety, depression, and other disorders [3,4]. With primary causes ranging from ovulation disorders to male factors and tubal diseases, infertility is influenced by various factors, including lifestyle choices and environmental conditions [5].

The role of trace elements, such as zinc, copper, and selenium, in reproductive health is increasingly recognized [6,7]. While copper has been linked to male fertility, understanding the precise relationship between trace elements and female fertility remains a developing area of research [8,9]. Early studies suggest a potential impact of nickel on pregnancy prevention, with conflicting findings regarding its effects on ovulation and ovarian function [10]. Further investigations are needed to elucidate the mechanisms and potential reproductive risks associated with nickel exposure, offering valuable insights into environmental factors affecting reproductive health. Our study aims to clarify the potential reproductive risks associated with nickel exposure, contributing valuable insights into the environmental factors affecting reproductive health. The primary objective of the study was to investigate the impact of urinary nickel levels on fertility.

## Materials and methods

This study leveraged the rich dataset provided by the National Health and Nutrition Examination Survey (NHANES), a program designed and conducted by the Centers for Disease Control and Prevention (CDC) to assess the health and nutritional status of adults and children in the United States. Our analysis specifically targeted sections of the NHANES dataset related to demographics, body measures, reproductive health, and urinary nickel levels, focusing on a subset of the population to explore potential environmental and physiological factors affecting fertility. The inclusion criteria were defined to consider women aged 18 to 35 years (a demographic typically associated with reproductive age) who had not undergone surgical procedures like hysterectomy or oophorectomy that directly impact fertility. Furthermore, to be included in the study, participants needed to have comprehensive data available on several critical variables, including fertility status, BMI, exposure to nickel, and detailed racial/ethnic background. The research protocol was approved by the Physician's Journal of Medicine Review Board located in Queens, New York, United States (approval number: 1223F128).

Variables

The investigation centered around the primary outcome variable of fertility status, which was determined based on participants' self-reported difficulties in achieving pregnancy. This binary outcome categorized women as fertile or infertile, excluding those with known surgical reasons for infertility, such as a hysterectomy or bilateral oophorectomy, to focus on potential unexplained or environmental causes of reduced fertility. Independent variables included in the analysis were carefully chosen to encompass a broad range of factors believed to influence fertility. Age was considered given its well-documented impact on reproductive potential, with fertility naturally declining as women age. Race/ethnicity was classified into several distinct categories to investigate any demographic patterns or disparities in fertility outcomes. BMI was analyzed as an indicator of overall health and its potential impact on fertility, with both underweight and obesity known to affect reproductive health. Lastly, urinary nickel concentration was examined as an environmental exposure variable, reflecting potential toxicological impacts on the reproductive system. Collectively, these variables were selected to provide a comprehensive understanding of the multifaceted influences on fertility among the study population.

Statistical analysis

The initial phase of statistical analysis involved the use of R statistical software (R Foundation for Statistical Computing, Vienna, Austria) for data preparation, including merging disparate data sources from NHANES and cleaning the dataset to ensure accuracy and consistency across variables. Descriptive statistics were then employed to characterize the study population, offering insights into the distribution and prevalence of fertility issues, as well as baseline levels of nickel exposure, BMI, and demographic compositions. The core analytical method, logistic regression, was applied to examine the relationship between the independent variables (age, race/ethnicity, BMI, and urinary nickel concentration) and the primary outcome of fertility status. This modeling approach allowed for the control of confounding factors and the estimation of odds ratios, providing a quantifiable measure of the strength of associations between fertility status and each of the predictors. Confidence intervals and p-values were also generated for each variable, offering statistical evidence of the reliability and significance of the observed relationships. 

## Results

The analysis encompassed data from the National Health and Nutrition Examination Survey (NHANES) focusing on a cohort of 7,839 women (Table 1). Specifically, attention was given to 1,404 (18%) women aged 18 to 35 years, a demographic chosen for its relevance to reproductive health studies. Within this age bracket, 901 (64%) women had undergone a hysterectomy, and 471 (34%) had a bilateral oophorectomy. The fertility status distribution was such that 880 (63%) women were identified as fertile, while 106 (8%) were categorized as infertile.

**Table 1:** Demographic characteristics NHANES: National Health and Nutrition Examination Survey

Variable	Count
Women in NHANES	7839
Women aged 18-35	1404
Women with hysterectomy	901
Women with bilateral oophorectomy	471
Infertile	106
Fertile	880

Racial categorization within the fertile group showed Non-Hispanic White women as the majority at 36.79%, with Non-Hispanic Black women following at 27.36% (Table 2). The representation of Mexican American and Other Hispanic women was comparatively lower at 9.43% (N =10) and 14.15% (N =15), respectively. Non-Hispanic Asian women constituted 8.49% (N = 9), and women of Other Races, including Multi-Racial, accounted for 3.77% (N =4) of the fertile group. The infertile group, however, presented a higher proportion of Mexican American women at 12.61% (N =111). Non-Hispanic White and Black women constituted 29.43% (N =259) and 28.64% (N =252), respectively, with Non-Hispanic Asian women and those of Other Races at 13.30% (N =117) and 6.14% (N =54).

**Table 2:** Comparison of fertility rates among different racial and ethnic groups

Race	Fertile women (%)	Infertile women (%)
Mexican American	10 (9.43%)	111 (12.61%)
Other Hispanic	15 (14.15%)	87 (9.89%)
Non-Hispanic White	39 (36.79%)	259 (29.43%)
Non-Hispanic Black	29 (27.36%)	252 (28.64%)
Non-Hispanic Asian	9 (8.49%)	117 (13.30%)
Other Race - Including Multi-Racial	4 (3.77%)	54 (6.14%)

Logistic regression analysis, controlling for race, BMI, and age, provided a nuanced understanding of this relationship. Nickel exposure, showed an inverse association with fertility, although not statistically significant (Coefficient = -0.176, Odds Ratio = 0.83862, p-value = 0.2401) (Table 3). The 95% confidence interval for nickel exposure ranged from -0.52285 to 0.03344, crossing the null value, which indicates a lack of statistical certainty in the effect size direction. Age emerged as a statistically significant factor in fertility status, with the analysis revealing that the likelihood of infertility increases with age (Coefficient = 0.13696, Odds Ratio = 1.14678, p-value = 0.00115). The 95% confidence interval for age did not overlap with the null value, supporting the reliability of this finding. 

**Table 3:** Predictive factors and odds ratios for fertility

Variable	Coefficient	Odds Ratio	p-value	CI Lower	CI Upper
Nickel	-0.176	0.83862	0.2401	-0.52285	0.03344
Race	-0.11956	0.88731	0.31103	-0.35871	0.10646
BMI	0.01657	1.01671	0.41401	-0.02444	0.05565
Age	0.13696	1.14678	0.00115	0.0574	0.22365

## Discussion

In the context of the considerable impact of infertility, which affects millions and leads to profound mental health issues, the role of trace elements in reproductive health emerges as a significant point of interest [6,7]. Essential elements like zinc, copper, and selenium are not only vital for overall health but play crucial roles in reproductive functions [7]. In pregnancies, the balance between oxidants and antioxidants, influenced by these nutrients, is crucial, as is their role in blood cell formation [7]. Copper, specifically, has been identified to have a clear connection with male fertility [8,9]. However, the understanding of how trace element levels influence female fertility is still developing, with much to be learned about the precise relationship between these micronutrients and the ability to conceive.

Early research, has identified some relationship between nickel and the prevention of pregnancy. When used as an intrauterine device in Holtzman rats, nickel wire was somewhat effective in preventing pregnancy when inserted early [10]. When examining the impact of nickel chloride (NiCl2) on pregnant Wistar rats, it was noted that there were changes in uterine tone and cellular damage at higher doses [11]. However, when exploring the mechanism of how nickel potentially disrupts the ovarian cycle, there is conflicting evidence. The same study examining nickel wire as an intrauterine device in Holtzman rats found no significant difference in the number of corpora lutea in the ovaries of rats [11]. In contrast, others have observed disturbed ovarian cycles and inhibited ovulation in rats exposed to NiSO4, suggesting a possible disruptive effect of nickel on ovarian function [12].

Further research has attempted to elucidate the role of nickel in pregnancy and reproductive processes. One study showed the effects of nickel nanoparticles and microparticles in rats, observing changes in ovarian tissue and inflammatory responses [13]. While another focused on the impact of NiCl2 on granulosa cells, noting decreased progesterone release and increased apoptotic cells at higher concentrations, hinting at possible mechanisms of nickel's reproductive toxicity [14]. Some data also imply a dose-response relationship with nickel exposure [15,16].

These studies noted declines in reproductive health markers in mice at higher doses of nickel, indicating that the extent of exposure may be a critical factor. Yet, other studies have shown no significant response in pregnancy outcomes in rats or humans. In a study evaluating the reproductive parameters in Long-Evans rats exposed to nickel chloride in drinking water, no major differences were found, except for a reduction in pup growth at a specific dose [17]. Similarly, other studies have shown slightly higher nickel levels in women with positive pregnancy tests, but the difference was not statistically significant [18]. Of the studies that did find a difference in nickel exposure and pregnancy, it was specific to polycystic ovary syndrome (PCOS) patients. This study found higher concentrations of nickel in a group of women with PCOS, with a significant association between nickel and decreased sex hormone-binding globulin [19].

This finding suggests a potential link between nickel exposure and hormonal changes. Further research has added layers of complexity to our understanding of trace elements and fertility. A study involving 45 women receiving in vitro fertilization treatments found elevated levels of urinary copper correlated with an increased number of oocytes and better embryo quality [20]. Moreover, investigations into the levels of copper within follicular fluid have yielded mixed results. In some cases, copper is linked to both positive and negative outcomes in women with endometriosis, a prevalent infertility condition [21,22]. However, most of the participants were either undergoing IVF or had known fertility issues, which poses a limitation in extending these results to all individuals, thus leaving some ambiguity in the connection between specific trace elements and fertility.

These studies collectively highlight the complexity of nickel's impact on reproductive health and the need for further investigation in human populations. By utilizing NHANES data to explore the correlation between urinary nickel levels and female fertility, our study aimed to clarify the potential reproductive risks associated with nickel exposure, contributing valuable insights into the environmental factors affecting reproductive health. Our findings seem to be consistent with current literature [23,24].

## Conclusions

While age was determined to be a significant factor in fertility outcomes, nickel exposure did not demonstrate a significant relationship with fertility status in the adjusted model. The study's results suggest that while there is an observed trend towards reduced fertility with higher nickel exposure, the association does not reach statistical significance when accounting for race, BMI, and age. These findings contribute to the ongoing discourse on environmental factors and reproductive health, underscoring the complexity of identifying clear causative agents in fertility research. Further studies are warranted to explore this association in greater depth, particularly considering potential interactions between nickel exposure and other unmeasured lifestyle or genetic factors.
